# Investigating synaptic plasticity in the crab *Cancer borealis* pyloric circuit and in a computational pyloric model network database

**DOI:** 10.1186/1471-2202-13-S1-P69

**Published:** 2012-07-16

**Authors:** Santiago Archila, Astrid A Prinz

**Affiliations:** 1 Department of Biology, Emory University, Atlanta, Georgia 30322, USA

## 

Homeostatic synaptic plasticity is a method employed by some neuronal networks to regulate their activity levels to stay within normal bounds. In this work, we investigate one particular synapse in the central-pattern-generating pyloric network of the crab *Cancer borealis* stomatogastric ganglion, and examine whether this synapse exhibits homeostatic plasticity.

The pyloric network of the crustacean stomatogastric ganglion consists of an intrinsically bursting pacemaker kernel – one anterior burster (AB) neuron and two pyloric dilator (PD) neurons that all share electrical connections – which rhythmically inhibits the non-intrinsically-bursting follower neurons – one lateral pyloric (LP) neuron and several pyloric (PY) neurons – to participate in a characteristic triphasic oscillatory pattern. The lone feedback to the pacemaker kernel is inhibitory and comes from the LP neuron. To test whether this synapse exhibits homeostatic plasticity, we measured the synaptic conductance of the pacemaker-feedback synapse (LP-to-PD) before, during, and after a strong perturbation of pyloric network activity. The perturbation consisted of either a depolarizing or hyperpolarizing voltage-clamp step of 25 mV from baseline (-60 mV) into one of the PD neurons and was considered a strong network-wide activity manipulation because many network activity features (such as cycle period, burst durations, duty cycles, etc.) were affected along with the post-synaptic PD membrane potential. We observe in several trials that the conductance of the LP-to-PD synapse changes significantly in response to the perturbation. Surprisingly, this conductance change seems not to oppose the PD membrane potential perturbation as we hypothesized. For instance, we hypothesized that the inhibitory feedback synapse to the pacemaker would strengthen if the PD neuron were depolarized in order to oppose the depolarization in a homeostatic manner through increased inhibition, but instead we observe a marked weakening of the feedback synapse in response to post-synaptic depolarization.

To further investigate what possible network activity effects could arise from this marked change in synaptic conductance, we turned to a previously described [1] computational pyloric network database. Using more than 4 million “pyloric-like” model networks, we analyzed the effect of a change in LP-to-PD synaptic conductance on several network-level activity features: cycle period, burst duration, duty cycle, delay, and phase. All network activity features were explored for both PD and LP. Following the computational analysis, we returned to the experimental results to investigate whether the observed change in synaptic conductance could be interpreted as homeostatically opposing disruptions in any of the network activity features. No such relationship was found, suggesting that the synaptic change we observed was not in the right direction for homeostasis for any of the network activity characteristics we examined.
